# A Texting- and Internet-Based Self-Reporting System for Enhanced Vaccine Safety Surveillance With Insights From a Large Integrated Health Care System in the United States: Prospective Cohort Study

**DOI:** 10.2196/58991

**Published:** 2024-10-11

**Authors:** Debbie E Malden, Julianne Gee, Sungching Glenn, Zhuoxin Li, Denison S Ryan, Zheng Gu, Cassandra Bezi, Sunhea Kim, Amelia Jazwa, Michael M McNeil, Eric S Weintraub, Sara Y Tartof

**Affiliations:** 1 Department of Research & Evaluation Kaiser Permanente Southern California Pasadena, CA United States; 2 Immunization Safety Office Centers for Disease Control and Prevention Atlanta, GA United States; 3 Kaiser Permanente Bernard J. Tyson School of Medicine Pasadena, CA United States

**Keywords:** digital health, survey participation, vaccine safety monitoring, COVID-19 vaccines, vaccine, vaccine safety, vaccine monitoring, text, text message, USA, US, surveillance, internet based, survey, monitoring, cohort study, self-reporting, vaccination, COVID-19 vaccination, medical records, text-based surveys, survey, surveys, surveillance system, data collection, disparity, vulnerable, EHR, electronic health records, mobile phone

## Abstract

**Background:**

SMS text messaging- and internet-based self-reporting systems can supplement existing vaccine safety surveillance systems, but real-world participation patterns have not been assessed at scale.

**Objective:**

This study aimed to describe the participation rates of a new SMS text messaging- and internet-based self-reporting system called the Kaiser Permanente Side Effect Monitor (KPSEM) within a large integrated health care system.

**Methods:**

We conducted a prospective cohort study of Kaiser Permanente Southern California (KPSC) patients receiving a COVID-19 vaccination from April 23, 2021, to July 31, 2023. Patients received invitations through flyers, SMS text messages, emails, or patient health care portals. After consenting, patients received regular surveys to assess adverse events up to 5 weeks after each dose. Linkage with medical records provided demographic and clinical data. In this study, we describe KPSEM participation rates, defined as providing consent and completing at least 1 survey within 35 days of COVID-19 vaccination.

**Results:**

Approximately, 8% (164,636/2,091,975) of all vaccinated patients provided consent and completed at least 1 survey within 35 days. The lowest participation rates were observed for parents of children aged 12-17 years (1349/152,928, 0.9% participation rate), and the highest participation was observed among older adults aged 61-70 years (39,844/329,487, 12.1%). Persons of non-Hispanic White race were more likely to participate compared with other races and ethnicities (13.1% vs 3.9%-7.5%, respectively; *P*<.001). In addition, patients residing in areas with a higher neighborhood deprivation index were less likely to participate (5.1%, 16,503/323,122 vs 10.8%, 38,084/352,939 in the highest vs lowest deprivation quintiles, respectively; *P*<.001). Invitations through the individual's Kaiser Permanente health care portal account and by SMS text message were associated with the highest participation rate (19.2%, 70,248/366,377 and 10.5%, 96,169/914,793, respectively), followed by email (19,464/396,912, 4.9%) and then QR codes on flyers (25,882/2,091,975, 1.2%). SMS text messaging–based surveys demonstrated the highest sustained daily response rates compared with internet-based surveys.

**Conclusions:**

This real-world prospective study demonstrated that a novel digital vaccine safety self-reporting system implemented through an integrated health care system can achieve high participation rates. Linkage with participants’ electronic health records is another unique benefit of this surveillance system. We also identified lower participation among selected vulnerable populations, which may have implications when interpreting data collected from similar digital systems.

## Introduction

Globally, there has been a rapid adoption of digital technologies, partly due to widespread access to mobile phones [1]. Mobile health (mHealth) has the power to revolutionize health care delivery and monitoring systems. Due to their ability to collect real-time self-reported symptom data from a large number of patients, mHealth systems have proven particularly useful in post licensure vaccine monitoring surveillance [2,3]. However, the broader applicability and significance of mHealth extends across multiple clinical domains, including chronic disease management, mental health support, and preventative care [4-7], underscoring the need to ensure their equitable implementation.

Despite this clear need, digital health care tools have been criticized for the underrepresentation of selected population groups, such as persons of different races or ethnicities or older age [8]. This phenomenon, known as the “digital divide” [9], impacts the generalizability of findings and risks exacerbating existing barriers to health care and health disparities [10,11]. Furthermore, in addition to enhancing equity, understanding and accounting for major drivers of participation also has important implications when interpreting data collected from mHealth systems. Without accounting for these systemic biases, epidemiological associations could be substantially altered, as demonstrated previously [12]. However, previous studies have lacked data with enough granularity within a closed system to identify population-level participation patterns.

These studies include several smartphone-based reporting systems for postvaccine reporting of adverse events (AEs) following immunizations [13-17]. However, since most systems rely on voluntary app-based smartphone enrollment, uptake has generally been low (often under 5% of vaccinated individuals) [3,18-22], contributing to a lack of representativeness. In addition, none of these studies have linked self-reported symptom data with individual-level electronic health record (EHR) data, limiting their ability to contextualize demographic or clinical information against that of the total vaccinated population and to validate clinical diagnoses. Evaluating participation in a similar digital system implemented across a large integrated health care system will improve the interpretation of data collected from SMS text messaging- and internet-based self-reporting systems and can inform the design of future systems. In January 2021, Kaiser Permanente Southern California (KPSC) developed the Kaiser Permanente Side Effect Monitor (KPSEM), a digital survey tool that allows patients to report on potential adverse events following COVID-19 vaccination. In this study, we aimed to assess KPSEM participation by demographic and clinical characteristics within a large integrated health care system.

## Methods

### Study Population

This prospective cohort study of all patients receiving COVID-19 vaccinations through KPSC from April 23, 2021, through July 31, 2023, monitored self-reported adverse events and solicited symptoms following vaccination.

### Data Collection

Patients receiving COVID-19 vaccinations at a KPSC facility could join the system online by scanning a QR code available on study flyers and posters using their smartphone devices. Except for SMS text messages, all study recruitment and communication materials in this study were branded with standard Kaiser Permanente institutional affiliations, including the study flyer, emails, and portal messages. Patients who did not sign up on the day of vaccination received an invitation by SMS text message, email, or a notification through their online Kaiser Permanente health care portal account. This included patients with Kaiser Permanente membership who were vaccinated by an external provider and identified through insurance claims databases and other integrated sources. Parents or guardians provided consent and submitted responses on behalf of their child under 18 years of age or their legal dependent. Initially, the Kaiser Permanente health care portal message center and SMS text message invitations were prioritized over email if patients had online accounts and valid contact information on file since these channels were found to result in higher participation rates during a pilot study (unpublished data). However, in December 2021, all health care portal communication was stopped due to concern of it interfering with care delivery messages, and SMS text messages became the prioritized channel of communication for initial invites. During the consent procedure, participants were asked whether they would prefer to complete the survey exclusively by SMS text message rather than online. After providing consent, patients were sent surveys inquiring about solicited AEs and symptoms at regular intervals according to their preferred contact method for up to 5 weeks after each dose; daily for the first week, alternate days for the second week, then weekly for 3 additional weeks. Survey wording and timing of survey questions are provided in Tables S1 and S2 in Multimedia Appendix 1. Upon receipt of additional vaccine doses, the survey cycle was restarted. Participants could actively withdraw from the survey at any point and would not be recontacted for subsequent doses. Survey information was subsequently combined with EHR data for access to patients’ demographic and clinical data across all care settings. Surveys were also made available in Spanish according to a patient’s on-file preference.

### Statistical Analysis

Selected characteristics were described among all patients who received a COVID-19 vaccination at KPSC over the study period and among those opting to participate in KPSEM. Participation was defined as completing at least 1 survey within 35 days of their vaccination. Selected clinical and demographic characteristics of interest included age, sex, race or ethnicity, socioeconomic status, and the presence of chronic comorbidities within 1 year before the date of first vaccination within the study period. Prespecified *ICD-10* (*International Statistical Classification of Diseases, Tenth Revision*) diagnosis codes were used to define comorbidities (Tables S3 and S4 in Multimedia Appendix 1). The neighborhood deprivation index (NDI) was used as an indicator of community-level socioeconomic status [23]. Race or ethnicity was categorized using mutually exclusive self-determined categories of non-Hispanic race (White, Black, or Asian), ethnicity (Hispanic), other (not within race or ethnicity groupings), or unknown race or ethnicity. Differences in survey participation statistics, survey reminder preferences (ie, SMS text message, email, or Kaiser Permanente portal message reminders), and self-reported solicited AEs and symptoms were described across selected demographic and clinical characteristics. Similarly, differences in participation (response rates and withdrawal rates over time) were described by separate recruitment and reminder channels, irrespective of demographic and clinical characteristics. Participant characteristics were compared using the chi-square test. All analyses were done with SAS Enterprise Guide statistical software (version 7.1) and R (version 4.3.0, R Foundation for Statistical Computing). Results were reported according to the updated CONSORT-EHEALTH (Consolidated Standards of Reporting Trials of Electronic and Mobile Health Applications and Online Telehealth) guidelines [24] and the CHERRIES (Checklist for Reporting Results of Internet E-Surveys) [25].

### Ethical Considerations

All study activities were reviewed by the Centers for Disease Control and Prevention (CDC) and completed in accordance with applicable Federal law and CDC policy. In addition, the study protocol was reviewed and approved by the KPSC institutional review board, which waived the requirement for informed consent (#12769).

## Results

### Study Population and Recruitment

**Figure 1:**
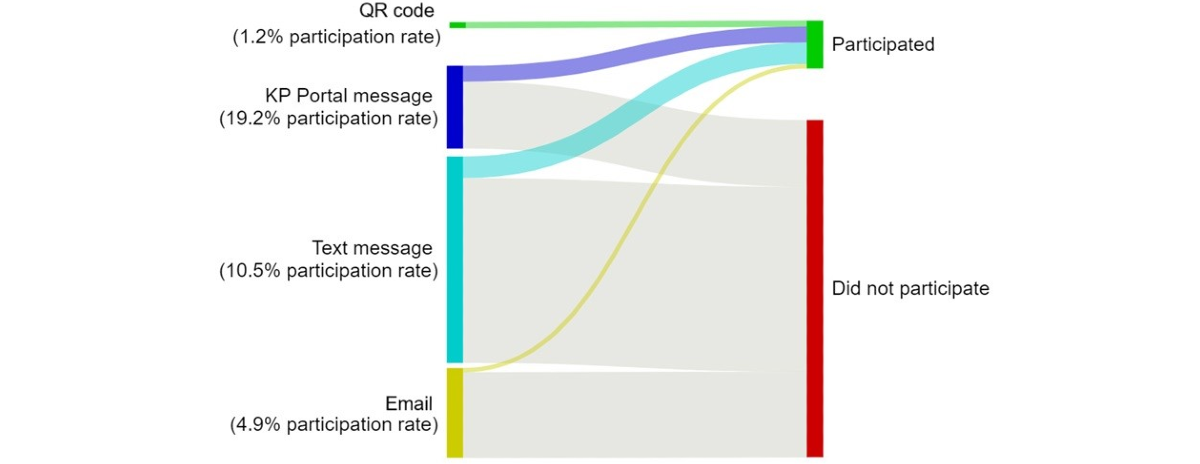
Participation rate by invitation channel among patients receiving a COVID-19 vaccination from April 23, 2021, to July 31, 2023. The participation rate was defined as follows: at least 1 documented report through Kaiser Permanente Side Effect Monitor within 35 days following receipt of vaccination ÷ total invited population × 100. KP: Kaiser Permanente.

Among 2,091,975 patients who were vaccinated between April 23, 2021, and July 31, 2023 (Table 1), 164,636 (7.9%) enrolled in the KPSEM system and completed at least 1 survey in the 35 days following the first dose of COVID-19 vaccination they received within the study period. This included patients who were vaccinated at KPSC facilities and KPSC members who received vaccination at non-KPSC facilities. Passive recruitment methods through patients scanning QR codes on flyers and posters at the time of vaccination enrolled only a small proportion of all vaccinated individuals (25,882/2,092,824, 1.2%; Figure 1 and Table S4 in Multimedia Appendix 1). Among persons contacted through their Kaiser Permanente health care portal account, 19.2% (70,248/366,377) joined and submitted at least 1 survey following their vaccination. Hence, this method was the most successful recruitment channel. SMS text message invitations resulted in a participation rate of 10.5% (96,169/914,793). Email invitations resulted in the lowest participation rate compared with the other active invitation channels (4.9%, 19,464/396,912). Among all individuals who received at least 1 digital invitation across all vaccine doses, participation was 13.1% (181,462/1,382,095; Table S4 in Multimedia Appendix 1).

**Table 1:** Characteristics of Kaiser Permanente Side Effect Monitor survey population receiving a COVID-19 vaccination from April 23, 2021, to July 31, 2023.

		All vaccinated patients, n				Participation rates^a^										
		First dose^b^	Second dose^b^	Third dose^b^	First dose ^b^				Second dose^b^				Third dose^b^			
					n (%)		*P* value^c^		n (%)		*P* value^c^		n (%)		*P* value^c^	
Total		2,091,975	1,087,240	352,086	164,636 (7.9)				56,268 (5.2)				26,054 (7.4)			
**Continuous KP^d^ membership^e^**								<.001				<.001				<.001
	Yes	1,738,566	962,252	333,236	148,141 (8.5)				53,545 (5.6)				25,462 (7.6)			
	No	353,409	124,988	18,850	16,495 (4.7)				2723 (2.2)				592 (3.1)			
**Age at vaccination (years)**								<.001				<.001				<.001
	0-4	35,594	28,895	12,467	2266 (6.4)				706 (2.4)				188 (1.5)			
	5-11	133,418	112,032	35,673	8001 (6)				4301 (3.8)				1381 (3.9)			
	12-17	152,928	92,191	23,789	1349 (0.9)				860 (0.9)				511 (2.1)			
	18-30	274,968	106,297	17,353	10,599 (3.9)				2061 (1.9)				519 (3.0)			
	31-40	265,379	99,747	16,253	19,032 (7.2)				4185 (4.2)				883 (5.4)			
	41-50	268,120	104,102	17,036	20,834 (7.8)				5346 (5.1)				1127 (6.6)			
	51-60	316,438	148,062	45,380	31,429 (9.9)				9831 (6.6)				4277 (9.4)			
	61-70	329,487	188,122	78,429	39,844 (12.1)				15,000 (8.0)				8356 (10.7)			
	71+	315,643	207,792	105,706	31,282 (9.9)				13,978 (6.7)				8812 (8.3)			
**Sex**								<.001				<.001				<.001
	Female	1,135,626	586,217	191,269	102,445 (9.0)				34,433 (5.9)				15,700 (8.2)			
	Male	956,349	501,023	160,817	62,191 (6.5)				21,835 (4.4)				10,354 (6.4)			
**Race and ethnicity**								<.001				<.001				<.001
	Hispanic	775,176	397,035	109,292	42,638 (5.5)				13,403 (3.4)				5080 (4.6)			
	Non-Hispanic Asian	253,029	134,827	49,590	16,675 (6.6)				5069 (3.8)				2285 (4.6)			
	Non-Hispanic Black	174,759	105,912	37,813	13,033 (7.5)				5197 (4.9)				2669 (7.1)			
	Other	117,099	59,152	18,778	8448 (7.2)				2729 (4.6)				1162 (6.2)			
	Non-Hispanic White	581,531	310,958	120,255	76,451 (13.1)				27,869 (9.0)				14,078 (11.7)			
	Unknown	190,381	79,356	16,358	7391 (3.9)				2001 (2.5)				780 (4.8)			
**NDI quintile^f^**								<.001				<.001				<.001
	1 (least deprived)	352,939	184,330	69,342	38,084 (10.8)				13,384 (7.3)				6855 (9.9)			
	2	448,763	232,809	80,942	42,978 (9.6)				14,744 (6.3)				6968 (8.6)			
	3	496,499	258,213	84,071	38,341 (7.7)				13,172 (5.1)				5997 (7.1)			
	4	465,142	242,381	71,573	28,461 (6.1)				9411 (3.9)				3997 (5.6)			
	5 (most deprived)	323,122	167,771	45,780	16,503 (5.1)				5499 (3.3)				2216 (4.8)			
	Unknown	5510	1736	378	269 (4.9)				58 (3.3)				21 (5.6)			

^a^Participation rate was calculated as: Participants with at least 1 documented report through KPSEM within 35 days following receipt of vaccination ÷ total vaccinated population × 100.

^b^COVID-19 vaccine dose is defined as a documented dose received within the defined study period, regardless of patient history of previous doses.

^c^Differences in participation rate were compared across demographic characteristics using the chi-square test for heterogeneity.

^d^KP: Kaiser Permanente.

^e^KP membership is defined as enrollment at least 1 year before vaccination and for 35 days following vaccination.

^f^Neighborhood Deprivation Index (NDI) was defined as the latest available NDI before COVID-19 vaccination.

### Patterns of Participation

**Figure 2:**
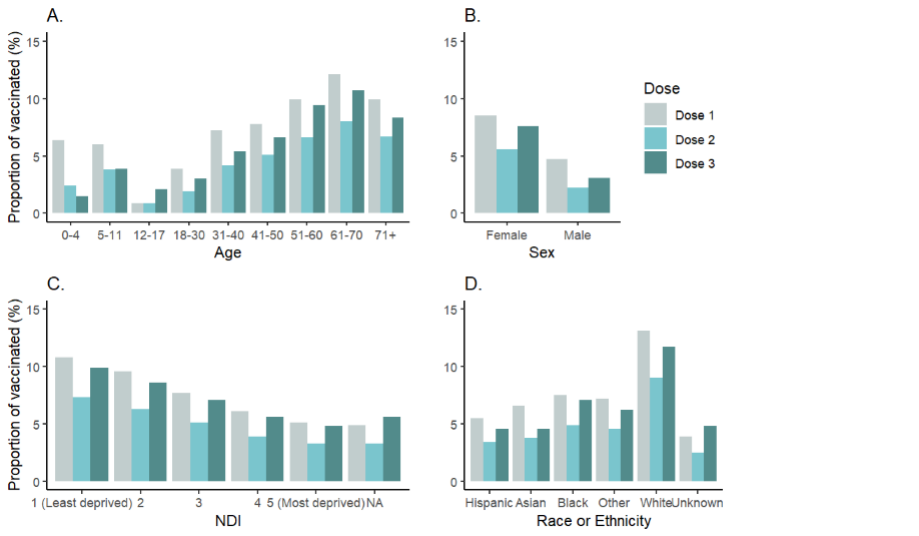
Participation rate by dose and demographic characteristics among patients receiving a COVID-19 vaccination from April 23, 2021, to July 31, 2023. Participation rate was defined as follows: participants with at least 1 documented report through Kaiser Permanente Side Effect Monitor within 35 days following receipt of vaccination/total vaccinated population x 100. NDI: Neighborhood Deprivation Index.

Females (102,445/1,135,626, 9%) were more likely to participate than males (62,191/956,349, 6.5%, *P*<.001; Table 1). Parents or guardians joining on behalf of their children demonstrated higher participation rates for younger children aged 0-4 years (2266/35,594, 6.4%) and 5-11 years (8001/133,418, 6%) than parents or guardians of children aged 12-17 years (1349/152,928, 0.9%; Figure 2). Low participation rates were also observed for young adults aged 18-30 years (10,599/274,968, 3.9%). The age group with the highest participation rates was adults aged 61-70 years (39,844/329,487, 12.1%). Participation also varied systematically by race or ethnicity and NDI; persons of non-Hispanic White race were more likely to participate compared with other races or ethnicities (13.1% vs 3.9%-7.5%, respectively, *P*<.001). Patients residing in areas with a higher NDI were less likely to participate (5.1%, 16,503/323,122 vs 10.8%, 38,084/352,939, in the highest vs lowest deprivation quintiles, respectively, *P*<.001). Overall, regardless of demographics, participation decreased for the second dose following dose 1 and then increased following the third dose (Figure 2).

**Figure 3:**
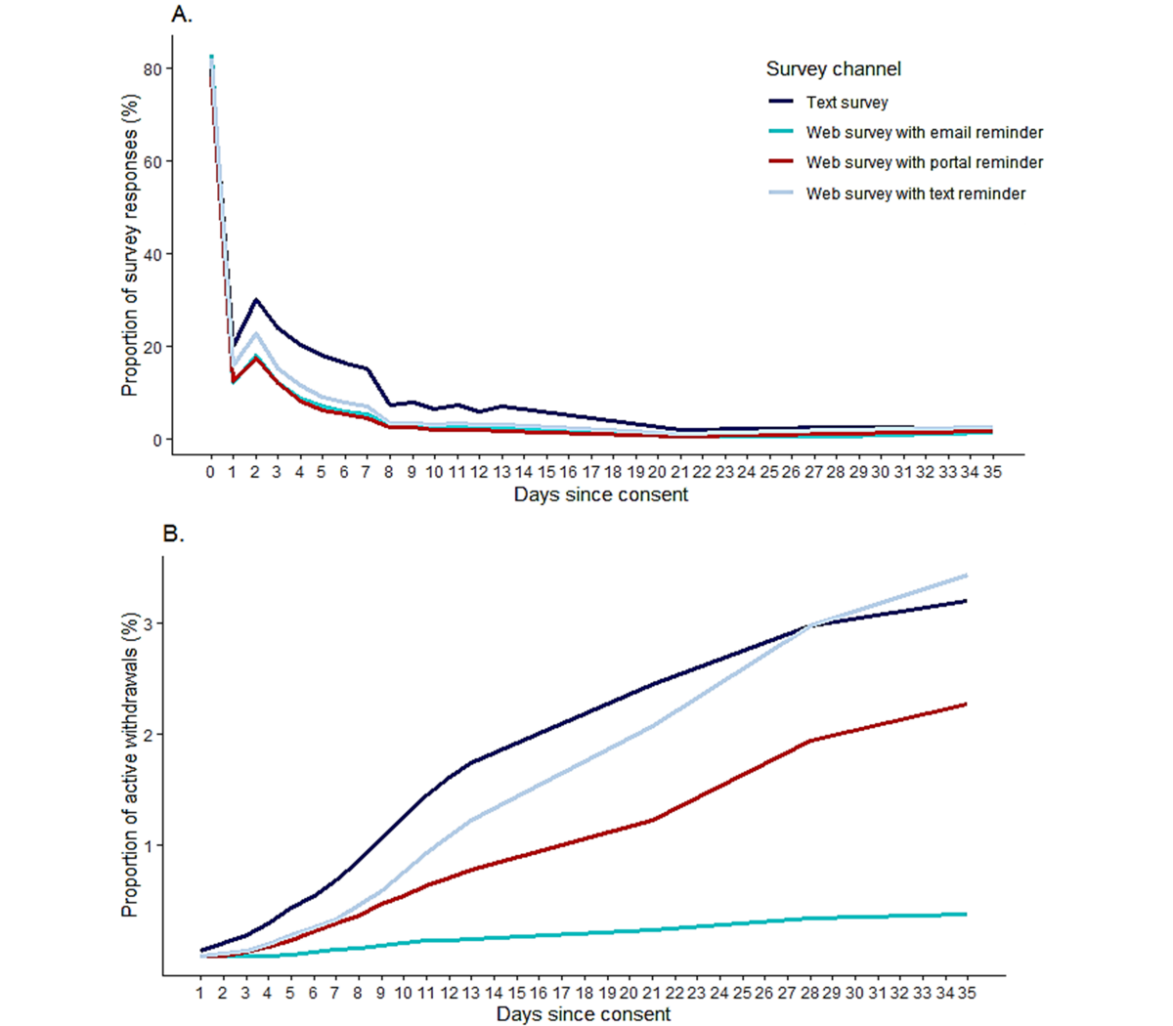
Rate of survey response (A) and active withdrawals (B) by day since consent and survey channel among Kaiser Permanente Side Effect Monitor participants receiving a COVID-19 vaccination from April 23, 2021, to July 31, 2023. Participation was defined as at least 1 documented report through Kaiser Permanente Side Effect Monitor within 35 days following receipt of COVID-19 vaccination.

Survey response rates dropped during the first week of survey notifications, from over 80% of participants across all invite channels on the day of consent (day 0) to below 30% of all enrolled participants on day 2 of the survey (Figure 3A). After the second day of the survey, the response rates consistently dropped through approximately week 3 of the survey and remained low until week 5 (<5% of enrolled participants). However, the sustained response rates differed by survey reminder channel, notably, SMS text message prompts and SMS text message surveys achieved consistently higher sustained responses compared with the other survey channels over 5 weeks. During the 35-day follow-up period, a cumulative proportion of around 3% of study participants withdrew from the study among those who were receiving SMS text message reminder notifications (Figure 3B). Withdrawals were less frequent over the same period among persons who opted to receive notifications through their Kaiser Permanente health care portal account (approximately 2%), and active withdrawals were lowest among persons receiving email reminders (<1%), although email reminders also had the lowest daily response rates (Figure 3A).

Initial reactions (local, systemic, and additional symptoms) were reported more frequently among persons who responded to multiple surveys during the 35-day follow-up period compared with participants who only responded to a single survey (Table S5 in Multimedia Appendix 1), as was the proportion reporting seeking medical care for symptoms; however, initial reports of reactions or solicited symptoms did not affect the likelihood of participation after subsequent vaccine doses (Table S6 in Multimedia Appendix 1).

## Discussion

### Principal Findings

Overall, these findings provide evidence for the feasibility of a digital self-reporting system as a timely, flexible, and scalable supplement to existing vaccine safety surveillance systems. We observed a high participation rate among vaccinated patients compared with other digital reporting systems deployed to a general US population. However, we also observed differences in participation across neighborhood-level socioeconomic characteristics and race or ethnicity, suggesting the existence of barriers to participation in these systems. If self-reporting digital health systems are to be used at scale, outreach efforts must be optimized to ensure that they accurately represent the underlying population. These findings also provide valuable information to improve the design of future digital self-reporting systems and to improve the interpretation and validity of the data collected through such systems.

There could be several reasons for the high participation rate of 30% observed in this study when compared with other digital vaccine safety self-reporting systems such as V-safe (CDC), which enrolled approximately 1-2% of all vaccinees in the United States during the national COVID-19 vaccination program [26]. First, owing to the integrated health care system setting (rather than a federal entity), our system may have had enhanced trust among participants [27,28]. An increase in awareness of the system by staff and patients could have also contributed to enhanced participation since a conscious effort was made to publicize the system through staff awareness sessions and internal communication platforms. Furthermore, recruitment invitations were sent to patients following receipt of multiple vaccine doses, further increasing their awareness of the system and the likelihood of participating with each new dose. In addition, by leveraging the expertise of trained digital teams, our system had many unique features designed to enhance survey usability and acceptance, such as the ability for enrollees to select their preferred survey methods (ie, SMS text message or internet-based surveys) and survey reminder preferences which may have improved participation and survey retention rates. Indeed, we observed that users had a strong preference for SMS text message surveys compared with other channels, and SMS text messages were associated with higher sustained survey response rates over 5 weeks. SMS text messages have proven to be an effective communication method to enhance trust and awareness of vaccine safety previously [29]. Furthermore, the preference for SMS text messaging–based systems over smartphone apps or targeted emails has been noted elsewhere [3,22], and most app-based reporting systems have demonstrated much lower participation rates compared with this study [3,18-20,22]. Previous work with patient usability surveys has identified barriers specifically related to smartphone apps, including lack of device memory, the need for software updates, and the additional time required to download apps [22]. Hence, not requiring a smartphone to participate in our system likely contributed to our observed high participation rates.

Akin to this study, other studies assessing the uptake of self-reporting systems for vaccine safety have identified the highest rates of participation among middle-aged adults and females [3,13,18-21,30]. In addition, we observed significant differences in participation by other demographic and socioeconomic characteristics not as well studied, most notably by NDI, where there was a clear inverse association between community-level socioeconomic deprivation and survey participation. Although general health care disparities predate the pandemic, they have been observed across many aspects of COVID-19–related care, including testing, vaccination, and severe outcomes, even in settings with no direct consumer health care costs such as prepaid insurance premiums [31]. As observed in this study, these disparities are also known to exist with respect to digital self-reporting systems [32]. However, although this area is less studied, encouragingly there is some evidence for reduced disparities for telehealth tools compared with in-person care [33]. The reasons underlying the observed disparities in digital system participation are unknown, but some have suggested that data literacy plays an important role [34]. Future research would benefit from understanding the barriers to participation in digital self-reporting systems, including eliminating gaps in digital literacy that could exacerbate health inequities.

As well as informing approaches for improved participation and potential targeted efforts for equitable uptake of future digital self-reporting systems, this study provides important insights that will aid in the interpretation of large-scale analysis using such data. This has not been feasible at scale previously due to the need for complete EHR data. For example, we identified that persons with immunocompromising conditions were more likely than the general vaccinated population to enroll in the system and, hence, may be overrepresented in similar studies. This is important when interpreting data from similar self-reporting vaccine safety monitoring systems since immunocompromised individuals may systematically differ with respect to reactions following vaccination [35]. In addition, most previous work has relied on enrollees inputting their demographic details and clinical information, which may adversely impact enrollment, reduce survey completion rates, and introduce data entry errors. In addition, one-third of smartphone-based vaccine safety app users in Germany reported that they found entering vaccination details difficult, often not knowing the vaccine’s name [19]. This exposure misclassification issue was also associated with certain patient demographics, such as advanced age, hence introducing systematic bias. Furthermore, another important finding from this study was that participation appeared to be slightly higher following a third vaccine dose compared with the second dose across most population subgroups studied. This could have been due to selection bias since third doses were not enforced to the same extent as the primary vaccine series, and hence they were more likely to be received by so-called “early adopters” or older or multimorbid populations, both of which are more likely to take part in research projects.

Data from previous self-reporting vaccine safety systems could be biased by the propensity of persons who have experienced reactions or AEs to be more motivated to engage in self-reporting studies. Interestingly, in this study, individuals who answered multiple surveys within 35 days following receipt of a vaccine were more likely to report local or systemic reactions on their initial report after the same dose and were also more likely to report seeking medical care for their reactions compared with participants who only answered the survey once. Therefore, reactions themselves may be a motivating factor underlying sustained survey participation and hence may lead to overestimations of symptom duration [36]. In contrast, while it was true that certain groups of participants were more likely to enroll after receipt of multiple vaccine doses in this study (parents of young children, persons of white race, etc.), baseline symptom reports did not differ greatly between persons who took part after only 1 dose compared with participation after multiple doses. Hence, we found no evidence that reporting reactogenicity to a previous vaccine dose influences participation after a subsequent vaccine dose. To date, no studies have investigated this phenomenon due to the need to access EHR for complete follow-up of a vaccinated cohort after multiple doses.

### Public Health Implications and Future Directions

Overall, our study findings demonstrated the ability of an SMS text messaging- and internet-based self-reporting system to complement other existing vaccine safety surveillance systems. The findings can also inform the future design of similar digital systems that seek to optimize the direct involvement of the public as empowered stakeholders in scientific research [37]. Furthermore, the population sociodemographic of persons likely to participate in such systems will inform the interpretation of future clinical studies using similar tools. Given the rapid expansion of digital health technology, understanding the potential for biased outputs should remain a priority for health care researchers [34].

### Limitations

There are some potential limitations to this study. First, the digital system was designed to operate almost entirely remotely, hence providing a real-world pragmatic overview of the scalability and uptake of our digital system. Consequently, patient usability was not formally assessed in a representative sample of users. However, previous studies incorporating usability surveys have generally lacked representativeness and were conducted among a small sample of participants due to feasibility constraints [22]. Second, although the long study period was a particular strength of this analysis, heightened media attention during the introduction of COVID-19 vaccines likely increased public awareness and possibly influenced participation during earlier months of the vaccine roll-out compared with later months. This could have explained the higher participation rate observed for older persons since they were eligible for vaccination earlier or among those invited through their Kaiser Permanente health care message center compared with other invitation channels because this contact route was halted after November 2021.

### Conclusion

In this study, an SMS text messaging- and internet-based self-reporting vaccine safety system enabled reactions to be reported by vaccinees in real time within a large and diverse managed care population. Hence, these findings demonstrated the feasibility of digital systems as timely and scalable methods that could supplement existing vaccine safety surveillance platforms. Our findings also emphasized the importance of implementing such a system through a trusted health care system for higher participation rates and potential clinical follow-up. However, we observed lower participation among selected vulnerable populations, demonstrating the existence of barriers to participation in digital reporting tools. If self-reporting digital tools are to be used in public health, their reach must be optimized to ensure that their implementation is equitable.

## Appendix

Multimedia Appendix 1 Additional information.

## Abbreviations

AE: adverse event
CDC: Centers for Disease Control and Prevention
CHERRIES: Checklist for Reporting Results of Internet E-Surveys (CHERRIES Checklist for Reporting Results of Internet E-Surveys
CONSORT-EHEALTH: Consolidated Standards of Reporting Trials of Electronic and Mobile Health Applications and Online Telehealth
EHR: electronic health record
*ICD-10*: *International Statistical Classification of Diseases, Tenth Revision*
KPSC: Kaiser Permanente Southern California
KPSEM: Kaiser Permanente Side Effect Monitor
mHealth: mobile health
NDI: Neighborhood Deprivation Index

## References

[1] Pew Research Center Mobile fact sheet.

[2] Zhou W, Pool V, Iskander JK, English-Bullard R, Ball R, Wise RP, Haber P, Pless RP, Mootrey G, Ellenberg SS, Braun MM, Chen RT (2003). Surveillance for safety after immunization: vaccine adverse event reporting system (VAERS)--United States, 1991-2001. MMWR Surveill Summ.

[3] Bota AB, Bettinger JA, Sarfo-Mensah S, Lopez J, Smith DP, Atkinson KM, Bell C, Marty K, Serhan M, Zhu DT, McCarthy AE, Wilson K (2023). Comparing the use of a mobile app and a web-based notification platform for surveillance of adverse events following influenza immunization: randomized controlled trial. JMIR Public Health Surveill.

[4] Gordon WJ, Landman A, Zhang H, Bates DW (2020). Beyond validation: getting health apps into clinical practice. NPJ Digit Med.

[5] Leigh JW, Gerber BS, Gans CP, Kansal MM, Kitsiou S (2022). Smartphone ownership and interest in mobile health technologies for self-care among patients with chronic heart failure: cross-sectional survey study. JMIR Cardio.

[6] Oudin A, Maatoug R, Bourla A, Ferreri F, Bonnot O, Millet B, Schoeller F, Mouchabac S, Adrien V (2023). Digital phenotyping: data-driven psychiatry to redefine mental health. J Med Internet Res.

[7] Willis VC, Thomas Craig KJ, Jabbarpour Y, Scheufele EL, Arriaga YE, Ajinkya M, Rhee KB, Bazemore A (2022). Digital health interventions to enhance prevention in primary care: scoping review. JMIR Med Inform.

[8] The Lancet Digital Health (2023). Digital health equity for older populations. Lancet Digit Health.

[9] Xiao A, Xu Z, Skare M, Qin Y, Wang X (2024). Bridging the digital divide: the impact of technological innovation on income inequality and human interactions. Humanit Soc Sci Commun.

[10] Ibrahim H, Liu X, Zariffa N, Morris AD, Denniston AK (2021). Health data poverty: an assailable barrier to equitable digital health care. Lancet Digit Health.

[11] Kc S, Tewolde S, Laverty AA, Costelloe C, Papoutsi C, Reidy C, Gudgin B, Shenton C, Majeed A, Powell J, Greaves F (2023). Uptake and adoption of the NHS App in England: an observational study. Br J Gen Pract.

[12] Pouwels K, Eyre DW, House T, Aspey B, Matthews PC, Stoesser N, Newton JN, Diamond I, Studley R, Taylor NGH, Bell JI, Farrar J, Kolenchery J, Marsden BD, Hoosdally S, Jones EY, Stuart DI, Crook DW, Peto TEA, Walker AS, COVID−19 Infection Survey Team (2024). Improving the representativeness of UK's national COVID-19 infection survey through spatio-temporal regression and post-stratification. Nat Commun.

[13] Antonelli M, Penfold RS, Merino J, Sudre CH, Molteni E, Berry S, Canas LS, Graham MS, Klaser K, Modat M, Murray B, Kerfoot E, Chen L, Deng J, Österdahl MF, Cheetham NJ, Drew DA, Nguyen LH, Pujol JC, Hu C, Selvachandran S, Polidori L, May A, Wolf J, Chan AT, Hammers A, Duncan EL, Spector TD, Ourselin S, Steves CJ (2022). Risk factors and disease profile of post-vaccination SARS-CoV-2 infection in UK users of the COVID symptom study app: a prospective, community-based, nested, case-control study. Lancet Infect Dis.

[14] Chapin-Bardales J, Gee J, Myers T (2021). Reactogenicity following receipt of mRNA-based COVID-19 vaccines. JAMA.

[15] Drury RE, O'Connor D (2021). Symptom study app provides real-world data on COVID-19 vaccines. Lancet Infect Dis.

[16] Houhamdi L, Fournier PE (2022). Smart apps for self-reporting clinical information. Lancet.

[17] Drury RE, O'Connor D (2021). Symptom study app provides real-world data on COVID-19 vaccines. Lancet Infect Dis.

[18] Menni C, Klaser K, May A, Polidori L, Capdevila J, Louca P, Sudre CH, Nguyen LH, Drew DA, Merino J, Hu C, Selvachandran S, Antonelli M, Murray B, Canas LS, Molteni E, Graham MS, Modat M, Joshi AD, Mangino M, Hammers A, Goodman AL, Chan AT, Wolf J, Steves CJ, Valdes AM, Ourselin S, Spector TD (2021). Vaccine side-effects and SARS-CoV-2 infection after vaccination in users of the COVID symptom study app in the UK: a prospective observational study. Lancet Infect Dis.

[19] Nguyen MTH, Krause G, Keller-Stanislawski B, Glöckner S, Mentzer D, Ott JJ (2021). Postmarketing safety monitoring after influenza vaccination using a mobile health app: prospective longitudinal feasibility study. JMIR Mhealth Uhealth.

[20] Nguyen MTH, Ott JJ, Caputo M, Keller-Stanislawski B, Klett-Tammen CJ, Linnig S, Mentzer D, Krause G (2020). User preferences for a mobile application to report adverse events following vaccination. Pharmazie.

[21] Rolfes L, Härmark L, Kant A, van Balveren L, Hilgersom W, van Hunsel F (2022). COVID-19 vaccine reactogenicity - a cohort event monitoring study in the Netherlands using patient reported outcomes. Vaccine.

[22] Wilson K, Atkinson KM, Westeinde J, Bell C, Marty K, Fergusson D, Deeks SL, Crowcroft N, Bettinger JA (2016). An evaluation of the feasibility and usability of a proof of concept mobile app for adverse event reporting post influenza vaccination. Hum Vaccin Immunother.

[23] Messer LC, Laraia BA, Kaufman JS, Eyster J, Holzman C, Culhane J, Elo I, Burke JG, O'Campo P (2006). The development of a standardized neighborhood deprivation index. J Urban Health.

[24] Eysenbach G, CONSORT-EHEALTH Group (2011). CONSORT-EHEALTH: improving and standardizing evaluation reports of web-based and mobile health interventions. J Med Internet Res.

[25] Eysenbach G (2004). Improving the quality of web surveys: the checklist for reporting results of internet E-surveys (CHERRIES). J Med Internet Res.

[26] Woo EJ, Gee J, Marquez P, Baggs J, Abara WE, McNeil MM, Dimova RB, Su JR (2023). Post-authorization safety surveillance of Ad.26.COV2.S vaccine: reports to the vaccine adverse event reporting system and v-safe, February 2021-February 2022. Vaccine.

[27] Pollard MS, Davis LM (2022). Decline in trust in the centers for disease control and prevention during the COVID-19 pandemic. Rand Health Q.

[28] Bogart LM, Ojikutu BO, Tyagi K, Klein DJ, Mutchler MG, Dong L, Lawrence SJ, Thomas DR, Kellman S (2021). COVID-19 related medical mistrust, health impacts, and potential vaccine hesitancy among black Americans living with HIV. J Acquir Immune Defic Syndr.

[29] Panickar R, Aziz Z, Mohd Sani N, Kamarulzaman A (2023). The use of technology in vaccine safety communication: a systematic review of randomised controlled trials. Patient Educ Couns.

[30] Hall VJ, Foulkes S, Saei A, Andrews N, Oguti B, Charlett A, Wellington E, Stowe J, Gillson N, Atti A, Islam J, Karagiannis I, Munro K, Khawam J, Chand MA, Brown CS, Ramsay M, Lopez-Bernal J, Hopkins S, SIREN Study Group (2021). COVID-19 vaccine coverage in health-care workers in England and effectiveness of BNT162b2 mRNA vaccine against infection (SIREN): a prospective, multicentre, cohort study. Lancet.

[31] Jefferson C, Watson E, Certa JM, Gordon KS, Park LS, D'Souza G, Benning L, Abraham AG, Agil D, Napravnik S, Silverberg MJ, Leyden WA, Skarbinski J, Williams C, Althoff KN, Horberg MA, NA-ACCORD Corona-Infectious-Virus Epidemiology Team (CIVET) (2022). Differences in COVID-19 testing and adverse outcomes by race, ethnicity, sex, and health system setting in a large diverse US cohort. PLoS One.

[32] Qian L, Sy LS, Hong V, Glenn SC, Ryan DS, Morrissette K, Jacobsen SJ, Xu S (2021). Disparities in outpatient and telehealth visits during the COVID-19 pandemic in a large integrated health care organization: retrospective cohort study. J Med Internet Res.

[33] Palzes VA, Chi FW, Metz VE, Sterling S, Asyyed A, Ridout KK, Campbell CI (2023). Overall and telehealth addiction treatment utilization by age, race, ethnicity, and socioeconomic status in California after COVID-19 policy changes. JAMA Health Forum.

[34] The Lancet Digital Health (2021). Power to the people. Lancet Digital Health.

[35] Chantasrisawad N, Puthanakit T, Tangsathapornpong A, Techasaensiri C, Phongsamart W, Suwanpakdee D, Jaruampornpan P, Sophonphan J, Suntarattiwong P, Chotpitayasunondh T, Study Team (2022). Immunogenicity and reactogenicity of mRNA BNT162b2 COVID-19 vaccine among thai adolescents with chronic diseases. Vaccines (Basel).

[36] Dos Santos G, Eckermann T, Martínez-Gómez X, Parra J, Nwoji U, Salamanca de la Cueva I (2023). Enhanced safety surveillance of GSK's quadrivalent seasonal influenza vaccine in Germany and Spain (2021/2022 season) using an electronic patient-reported outcome system for vaccine safety remote monitoring. Influenza Other Respir Viruses.

[37] Cohen AB, Mathews SC, Dorsey ER, Bates DW, Safavi K (2020). Direct-to-consumer digital health. Lancet Digit Health.

