# Supplementary material for: A Texting- and Internet-Based Self-Reporting System for Enhanced Vaccine Safety Surveillance With Insights From a Large Integrated Health Care System in the United States: Prospective Cohort Study
Source: JMIR Mhealth Uhealth. 2024 Oct 11;12:e58991. doi: 10.2196/58991 (PMC11512128; doi:10.2196/58991)
Supplement: Multimedia Appendix 1 [file mhealth_v12i1e58991_app1.pdf]

Table S1. Survey wording for web-based and text message-based surveys in persons aged >3 years receiving a COVID-19 vaccination April 23, 2021 – July 31, 2023.

Question timing	Online secure link survey	Text message survey	Timing
Survey Q1: Prior COVID-19 status (only asked one time in first ever survey)	Have you ever had a positive COVID-19 test or been told by a health care provider that you had COVID-19?<br>[Check boxes - Yes & No - automatically continues to next page]	Have you ever had a positive COVID-19 test or been told by a health care provider that you had COVID-19? Reply YES if you have, NO if you have not. Reply END to opt-out, or HELP for help.	Day 1
Survey Q2: Local reactions	Since getting your COVID-19 vaccine, have you experienced any of the following side effects at or near where you got the shot? Please select all that apply. [Check boxes - Pain, Redness, Itching, Swelling, No symptoms]	Since getting your COVID-19 vaccine, have you experienced any of the following side effects at or near where you got the shot?<br>1. Pain<br>2. Redness<br>3. Swelling<br>4. Itching<br>5. None of the above<br>Reply with the numbers corresponding to the side effects you've experienced. For example: 1, 3.<br>Reply END to opt-out, or HELP for help.	Weeks 1-2
Survey Q3: Systemic reactions I	Since getting your COVID-19 vaccine, have you experienced any of the following side effects? Please select all that apply. [Check boxes - Fever, Chills, Fatigue or tiredness, Joint paint or body or muscle aches, Headache, Nausea or vomiting, Rash (not including injection site), No symptoms]	Since getting your COVID-19 vaccine, have you experienced any of the following side effects?<br>1. Fever<br>2. Chills<br>3. Fatigue or tiredness<br>4. Joint pain or body or muscle aches<br>5. Headache<br>6. Nausea or vomiting<br>7. Rash, not including the injection site<br>8. None of the above<br>Reply with the numbers corresponding to the side effects you've experienced. For example: 1, 3.<br>Reply END to opt opt-out, or HELP for help.	Weeks 1-2
Survey Q4: Systemic reactions II	Since getting your COVID-19 vaccine, have you experienced any of the following side effects? Please select all that apply. [Check boxes - Persistent fast heart rate, Lightheadedness Painful, burning, or tingling rash (shingles); Menstrual cycle changes, if applicable; Chest Pain; Other symptoms; No symptoms]	Since getting your COVID-19 vaccine, have you experienced any of the following side effects?<br>1. Persistent fast heart rate<br>2. Lightheadedness<br>3. Painful, burning, or tingling rash (shingles)<br>4. Menstrual cycle changes, if applicable<br>5. Chest Pain<br>6. Other symptoms<br>7. None of the above<br>Reply with the numbers corresponding to the side effects you've experienced. For example: 1, 3.<br>Reply END to opt-out, or HELP for help.	All surveys, weeks 1-5
Survey Q5: Medical care (if yes to previous Qs)	Did any of these symptoms cause you to seek medical care?<br>[Check boxes - Yes & No - automatically continues to next question]	Did any of these symptoms cause you to seek medical care?<br>Reply YES if you have, NO if you have not.<br>Reply END to opt-out, or HELP for help.	All surveys, weeks 1-5

Table S2. Survey wording for web-based and text message-based surveys in persons aged 6 months – 3 years receiving a COVID-19 vaccination April 23, 2021 – July 31, 2023.

Question timing	Online secure link survey	Text message survey	Timing
Survey Q1: Prior COVID-19 status (only asked one time in first ever survey)	Have you ever had a positive COVID-19 test or been told by a health care provider that you had COVID-19?<br>[Check boxes - Yes & No - automatically continues to next page]	Have you ever had a positive COVID-19 test or been told by a health care provider that you had COVID-19? Reply YES if you have, NO if you have not. Reply END to opt-out, or HELP for help.	Day 1
Survey Q2: Local reactions	Since getting their COVID-19 vaccine, has your child/dependent experienced any of the following side effects at or near where you got the shot? Please select all that apply. [Check boxes - Pain, Redness, Swelling/hardness, Groin or underarm swelling/tenderness, None of the above]	Since getting their COVID-19 vaccine, has your child/dependent experienced any of the following side effects at or near where you got the shot?<br>1. Pain<br>2. Redness<br>3. Swelling/hardness<br>4. Groin or underarm swelling/tenderness<br>5. None of the above<br>Reply with the numbers corresponding to the side effects you've experienced. For example: 1, 3.<br>Reply END to opt-out, or HELP for help.	Weeks 1-2
Survey Q3: Systemic reactions I	Since getting their COVID-19 vaccine, has your child/dependent experienced any of the following side effects?<br>Please select all that apply. [Check boxes - Fever, Sleepiness , Irritability/crying, Loss of appetite, Vomiting, Diarrhea, Rash, not including the injection site, Other, None of the above]	Since getting their COVID-19 vaccine, has your child/dependent experienced any of the following side effects?<br>1. Fever<br>2. Sleepiness<br>3. Irritability/crying<br>4. Loss of appetite<br>5. Vomiting<br>6. Diarrhea<br>7. Rash, not including the injection site<br>8. Other<br>9. None of the above<br>Reply with the numbers corresponding to the side effects you've experienced. For example: 1, 3.<br>Reply END to opt opt-out, or HELP for help.	Weeks 1-5
Survey Q5: Medical care (if yes to previous Qs)	Did any of these symptoms cause you to seek medical care for your child or dependent?<br>[Check boxes - Yes & No - automatically continues to next question]	Did any of these symptoms cause you to seek medical care for your child or dependent?<br>Reply YES if you have, NO if you have not.<br>Reply END to opt-out, or HELP for help.	All surveys, weeks 1-5

Table S3. Clinical characteristics and healthcare seeking behavior of Kaiser Permanente Side Effect Monitoring system survey participants receiving a COVID-19 vaccination April 23, 2021 – July 31, 2023.

	All vaccinated patients			Participation [illegible]		
	1st Dose[b]	2nd Dose[b]	3rd Dose[b]	1st Dose[b]		2nd D[illegible]
				N (%)	P value[4]	N (%)
**Total**	1,738,566	962,252	333,236	148,141 (8.5%)		53,545 (5.6%)
**At least one SARS-CoV-2 test in the year prior**					<.0001	
Yes	839,466	499,747	179,649	78,805 (9.4%)		30,552 (6.1%)
No	899,100	462,505	153,587	69,336 (7.7%)		22,993 (5.0%)
**Outpatient visits in the year prior**					<.0001	
0	2,082	285	7	117 (5.6%)		8 (2.8%)
1-4	174,347	35,423	351	8,905 (5.1%)		723 (2.0%)
>4	1,562,137	926,544	332,878	139,119 (8.9%)		52,814 (5.7%)
**Received influenza vaccination in the year prior**					<.0001	
Yes	1,134,930	688,046	272,433	113,362 (10.0%)		45,107 (6.6%)
No	603,636	274,206	60,803	34,779 (5.8%)		8,438 (3.1%)
**Insurance status**					<.0001	
State-subsidized	173,670	104,781	32,046	8,235 (4.7%)		3,208 (3.1%)
Not subsidized	1,562,954	856,293	300,872	139,782 (8.9%)		50,299 (5.9%)
No coverage at vaccination	1,942	1,178	318	124 (6.4%)		38 (3.2%)
**BMI, N (%)**					<.0001	
**<18**	136,301	105,911	38,853	7,467 (5.5%)		3,724 (3.5%)
18-25	575,476	313,894	108,269	46,823 (8.1%)		16,913 (5.4%)
26-30	478,495	253,605	89,427	43,271 (9.0%)		15,054 (5.9%)
31-40	401,992	210,541	71,676	37,931 (9.4%)		13,348 (6.3%)
>40	82,278	42,721	13,324	8,852 (10.8%)		3,089 (7.2%)
Unknown	64,024	35,580	11,687	3,797 (5.9%)		1,417 (4.0%)
**Comorbidity, N (%)**						
Type II Diabetes	253,652	156,915	68,140	21,787 (8.6%)	<.0001	8,485 (5.4%)
Stroke	17,210	10,613	4,722	1,310 (7.6%)	<.0001	526 (5.0%)
Chronic Heart Failure	8,634	5,761	2,722	810 (9.4%)	0.744	369 (6.4%)
Chronic Kidney Disease	104,648	70,769	36,277	9,662 (9.2%)	<.0001	4,382 (6.2%)
Chronic Heart Failure	33,638	21,550	10,219	2,742 (8.2%)	<.0001	1,109 (5.1%)
COPD	29,222	19,073	9,667	3,101 (10.6%)	0.0006	1,332 (7.0%)
Immunocompromised††	351,985	219,262	98,038	40,477 (11.5%)	<.0001	17,024 (7.8%)

[a]Participants with at least one documented report via KPSEM within 35 days following receipt of vaccination/ total vaccinated population.

[b]COVID-19 vaccine dose defined as a documented dose received within the defined study period, regardless of patient history of prior doses.

[c]Differences in participation rate were compared across demographic characteristics using the chi-square test for heterogeneity.

Table S4. Characteristics of Kaiser Permanente Side Effect Monitoring system survey participants receiving a COVID-19 vaccination April 23, 2021 – July 31, 2023, by type of invitation channel.

	Vaccinated Patients	QRC	KP.org message center		SMS		EMAIL		C
		Participated[a]	Invited	Participated[a]	Invited	Participated[a]	Invited	Participated[a]	
**Overall**	2,092,824	25,882	366,377	70,248	914,793	96,169	396,912	19,464	1.
**KP membership**[2]									
Yes	1,763,897	23,597	339,976	66,928	803,575	85,868	349,377	16,983	1.
No	328,927	2,285	26,401	3,320	111,218	10,301	47,535	2,481	0.
**Sex**									
Female	1,135,904	16,120	199,632	42,917	498,748	60,492	212,815	12,185	1.
Male	956,920	9,762	166,745	27,331	416,045	35,677	184,097	7,279	1.
**Age at vaccination**									
0-4	34,386	332	470	3	22,683	2,349	9,638	203	1.
5-11	128,192	1,019	21,468	3,338	69,272	3,359	30,628	464	0.
12-17	155,548	735	32,377	2,704	56,970	1,720	13,281	211	0.
18-30	276,035	1,699	36,320	3,511	102,390	7,265	46,467	1,513	0.
31-40	264,269	2,979	42,362	6,982	96,788	11,688	48,326	2,627	1.
41-50	265,507	3,249	42,859	7,232	101,291	12,751	48,168	2,724	1.
51-60	312,402	4,672	47,889	10,286	138,133	19,286	65,312	3,804	1.
61-70	324,982	5,861	64,632	17,215	159,944	21,682	67,927	4,097	1.
71+	331,503	5,336	78,000	18,977	167,322	16,069	67,165	3,821	1.
**Race/Ethnicity**									
Hispanic	776,280	6,803	119,486	14,684	339,732	28,260	147,182	5,398	0.
Non-Hispanic Asian	253,260	2,574	50,240	7,603	121,143	9,237	54,034	1,826	1.
Non-Hispanic Black	174,826	2,211	31,107	5,537	80,406	8,360	35,240	1,536	1.
Other	117,318	1,322	20,831	3,575	51,734	4,985	23,465	994	1.
Non-Hispanic White	580,514	11,742	127,717	36,354	260,730	39,994	110,238	8,409	2.
Unknown	190,626	1,230	16,996	2,495	61,048	5,333	26,753	1,301	0.
**Insurance status**									
State-subsidized	202,170	1,646	30,226	3,737	91,413	6,615	38,754	1,286	0.
Not subsidized	1,741,920	23,653	330,165	66,018	781,991	86,685	343,374	17,427	1.
No coverage	148,734	583	5,986	493	41,389	2,869	14,784	751	0.
**NDI quintile**[3]									
1 (Least deprived)	353,079	5,945	74,118	18,465	158,684	20,111	67,181	4,155	1.
2	449,810	6,710	86,606	19,106	199,328	23,880	86,834	5,060	1.
3	496,873	6,029	86,364	15,846	218,917	23,023	95,332	4,488	1.
4	464,800	4,537	73,513	10,909	199,431	18,035	87,355	3,558	1.
5 (Most deprived)	322,852	2,614	45,299	5,829	136,761	10,946	59,531	2,167	0.
Unknown	5,410	47	477	93	1,672	174	679	36	0.

[a]Participants with at least one documented report via KPSEM within 35 days following receipt of vaccination/ total vaccinated population.

[b]KP membership defined as enrollment at least 1 year prior to vaccination and for 35 days following vaccination.

[c]Neighborhood Deprivation Index (NDI) was defined as the latest available NDI prior to COVID-19 vaccination.

Table S5. Characteristics of Kaiser Permanente Side Effect Monitor participants receiving a COVID-19 vaccination April 23, 2021 – July 31, 2023 with at least one documented survey response during the 35 days following vaccination.

	Responded (signed up and answered the survey) ONLY ONCE in 35 days[a]		Responded (signed up and answered the survey) more than once in 35 days[b]	
	**N**	**%***	**N**	**%***
**Overall**	52,524	100.0%	169,402	100.0%
**KP membership**				
Yes	45,984	87.5%	153,625	90.7%
No	6,540	12.5%	15,777	9.3%
**Sex**				
Female	32,464	61.8%	106,116	62.6%
Male	20,060	38.2%	63,286	37.4%
**Age at vaccination**				
4-11	1,904	3.6%	7,645	4.5%
12-17	1,207	2.3%	4,011	2.4%
18-30	4,714	9.0%	11,003	6.5%
31-60	22,872	43.6%	73,062	43.1%
61-70	11,969	22.8%	40,666	24.0%
71+	9,858	18.8%	33,015	19.5%
**Race/Ethnicity**				
Hispanic	15,025	28.6%	42,865	25.3%
Asian	5,996	11.4%	16,435	9.7%
Black	4,226	8.0%	14,132	8.3%
Other	2,870	5.5%	8,483	5.0%
White	21,450	40.8%	79,437	46.9%
Unknown	2,957	5.6%	8,050	4.8%
**Received flu vaccination in the past year**				
Yes	37,859	72.1%	124,198	73.3%
No	14,665	27.9%	45,204	26.7%
**Symptom reports**				
Any	33,787	64.3%	131,173	77.4%
Local symptoms	29,083	55.4%	109,295	64.5%
Systemic symptoms	27,209	51.8%	109,121	64.4%
Additional symptoms	11,853	22.6%	64,105	37.8%
Medical care seeking	1,019	1.9%	4,400	2.6%

[a] Patients aged≥4 consented and participated on the first day (day 0) and first day only.

[b] Patients aged≥4 consented and participated on the first day (day 0) and any of the subsequent day(s) up to 35 days following vaccination.

Table S6. Characteristics of Kaiser Permanente Side Effect Monitor survey participants aged 4 years and above who received two or more COVID-19 vaccine doses during April 23, 2021 – July 31, 2023 by the number of responses.

	Responded only once after one dose[a]		Responded after at least two doses[b]	
	N	%*	N	%*
**Overall**	44,861	100.0%	45,793	100.0%
**KP membership**				
Yes	41,455	92.4%	43,209	94.4%
No	3,406	7.6%	2,584	5.6%
**Sex**				
Female	27,723	61.8%	27,644	60.4%
Male	17,138	38.2%	18,149	39.6%
**Age at vaccination**				
4-11	2,720	6.1%	4,054	8.9%
12-17	750	1.7%	333	0.7%
18-30	2,696	6.0%	1,307	2.9%
31-60	19,847	44.2%	14,519	31.7%
61-70	10,623	23.7%	13,734	30.0%
71+	8,225	18.3%	11,846	25.9%
**Race/Ethnicity**				
Hispanic	12,276	27.4%	9,840	21.5%
Asian	4,958	11.1%	4,089	8.9%
Black	3,800	8.5%	4,233	9.2%
Other	2,397	5.3%	2,129	4.6%
White	19,568	43.6%	24,015	52.4%
Unknown	1,862	4.2%	1,487	3.2%
**Received flu vaccination in the past year**				
Yes	34,074	76.0%	37,524	81.9%
No	10,787	24.0%	8,269	18.1%
**Symptom reports**				
Any	39,409	87.8%	40,188	87.8%
Local symptoms	33,062	73.7%	33,021	72.1%
Systemic symptoms	32,481	72.4%	31,969	69.8%
Additional symptoms	17,412	38.8%	17,796	38.9%
Medical care	1,207	2.7%	971	2.1%

[a] Patients aged≥4 with two or more survey cycles consented and participated only once
[b] Patients aged≥4 with two or more survey cycles consented and participated more than once
